# The putative oligosaccharide translocase SypK connects biofilm formation with quorum signaling in *Vibrio fischeri*

**DOI:** 10.1002/mbo3.199

**Published:** 2014-09-25

**Authors:** Tim Miyashiro, Dane Oehlert, Valerie A Ray, Karen L Visick, Edward G Ruby

**Affiliations:** 1 Department of Biochemistry and Molecular Biology, Eberly College of Science, The Pennsylvania State UniversityUniversity Park, Pennsylvania, 16802; 2 Department of Medical Microbiology and Immunology, University of Wisconsin School of Medicine and Public HealthMadison, Wisconsin, 53706; 3 Department of Microbiology and Immunology, Loyola University Medical CenterMaywood, Illinois, 60153

**Keywords:** Biofilm, gene regulation, molecular genetics, quorum sensing

## Abstract

Quorum signaling (QS) describes how bacteria can use small signaling molecules (autoinducers) to coordinate group-level behaviors. In *Vibrio fischeri,* QS is achieved through a complex regulatory network that ultimately controls bioluminescence, motility, and host colonization. We conducted a genetic screen focused on *qrr1*, which encodes a small regulatory RNA that is necessary for the core quorum-signaling cascade to transduce autoinducer information into cellular responses. We isolated unique mutants with a transposon inserted into one of two genes within the *syp* locus, which is involved in biofilm formation. We found that overexpression of *sypK*, which encodes a putative oligosaccharide translocase, is sufficient to activate *qrr1*, and, in addition, this effect appears to depend on the kinase activity of the sensor LuxQ. Consistent with the established model for QS in *V. fischeri*, enhanced expression of *qrr1* by the overexpression of *sypK* resulted in reduced bioluminescence and increased motility. Finally, we found that induction of the *syp* locus by overexpression of *sypG* was sufficient to activate *qrr1* levels. Together, our results show how conditions that promote biofilm formation impact the quorum-signaling network in *V. fischeri*, and further highlight the integrated nature of the regulatory circuits involved in complex bacterial behaviors.

## Introduction

Quorum signaling (QS) describes the process that enables a bacterium to sense and respond to other bacteria (Fuqua et al. 2001; Ng and Bassler 2009). The cell-signaling systems associated with QS depend on the synthesis and detection of signaling molecules, called autoinducers. For many bacterial species, these QS systems enable the coordination of population-level responses through gene regulation. Because autoinducer concentrations are often proportional to cell density, the responses to QS are also traditionally characterized according to cell density. However, this correlation can be disrupted by additional signaling components that occur downstream of the autoinducer receptor(s) within the regulatory network. Therefore, studies aimed to identify such inputs are critical for understanding how QS systems function in nature.

**Figure 1:**
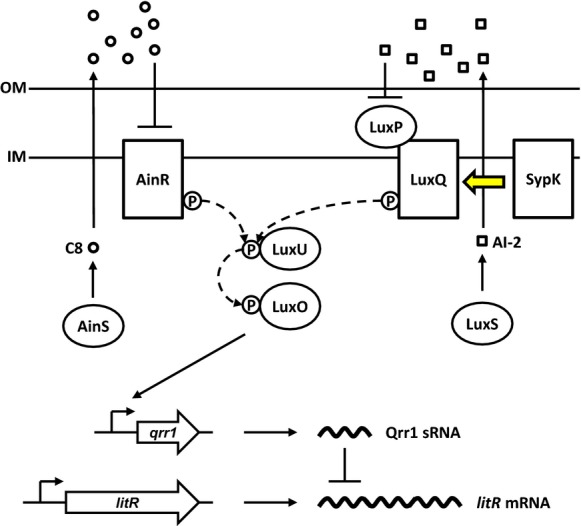
Model of the core quorum-signaling (QS) system in *Vibrio fischeri*. The outputs of the QS systems AinS/AinR and LuxS/LuxP/LuxQ converge on the LuxU/LuxO phosphorelay. Phosphorylated LuxO activates transcription of the small regulatory RNA Qrr1 that posttranscriptionally represses *litR*, which encodes the transcription factor LitR. In this study, we show that SypK modulates QS by affecting the kinase activity of LuxQ (indicated by the yellow arrow).

*Vibrio fischeri* is a marine bacterium that uses QS to regulate a multitude of cellular processes, including bioluminescence, motility, and colonization of its natural host, the Hawaiian bobtail squid, *Euprymna scolopes* (Nyholm and McFall-Ngai 2004; Miyashiro and Ruby 2012; Stabb and Visick 2013; Verma and Miyashiro 2013). The LuxR-LuxI QS system directly regulates the *lux* genes, which encode the light-producing enzyme luciferase and several proteins involved in light production and other activities. LuxR is a transcription factor activated by the autoinducer *N-*3-oxohexanoyl-homoserine lactone (3-oxo-C6), which is produced by the synthase LuxI. *V. fischeri* possesses additional QS systems that converge on a signaling cascade that, unlike the LuxR-LuxI system, is conserved among all *Vibrionaceae* members (Milton 2006). At its core is a phosphorelay composed of the histidine phosphotransfer protein LuxU and the response regulator LuxO (Fig. 1). Based primarily on the studies of the analogous phosphorelay in *Vibrio harveyi*, LuxU is predicted to become phosphorylated on a conserved histidine residue by the kinases AinR and LuxQ under conditions of low autoinducer concentrations, for example, low cell density (Freeman and Bassler 1999a, 1999b; Ray and Visick 2012). Whereas AinR appears to serve as the receptor for the AinS-derived autoinducer *N-*octonoyl-homoserine lactone (C8) (Gilson et al. 1995; Kimbrough and Stabb 2013), the periplasmic protein LuxP is thought, based on work in *V. harveyi*, to bind to the furanosyl borate diester, autoinducer-2 (AI-2), which modulates the kinase activity of LuxQ toward LuxU (Neiditch et al. 2005, 2006). Upon phosphorylation, LuxU is predicted to donate the phosphoryl group to a conserved aspartic acid residue of LuxO, which can then activate transcription of *qrr1* (Miyashiro et al. 2010). The RNA chaperone Hfq assists the small regulatory RNA (sRNA) Qrr1 in the posttranscriptional repression of LitR, a global transcription factor that regulates motility, host colonization factors, and bioluminescence (Fidopiastis et al. 2002; Miyashiro et al. 2010; Cao et al. 2012). The net effect of the integrated QS systems is that under high cell density (i.e*.,* in the presence of autoinducers) LuxO becomes de-phosphorylated, which leads to low *qrr1* expression and the ability of *V. fischeri* to fully activate the *lux* genes.

Within the past decade, *V. fischeri* has also become a useful model organism to explore the genetic determinants for developing biofilms, which are elaborate structures that bacterial populations or communities can produce to associate with surfaces and each other (Visick 2009; Yildiz and Visick 2009). By synthesizing and exporting various exopolysaccharides and other molecules (Flemming et al. 2007), bacteria can remain attached to a surface and sheltered from unpredictable and potentially stressful environments. Wild-type *V. fischeri* does not produce a substantial biofilm under standard laboratory conditions. However, activation of a cluster of 18 genes that comprise the *syp* locus (Yip et al. 2005) confers phenotypes associated with biofilms, such as the ability to form wrinkled colonies on solid agar surfaces (Yip et al. 2006; Hussa et al. 2008). QS was recently shown to impact the dynamics of *syp*-mediated biofilm development as mutants containing an insertion in *luxQ* were delayed in wrinkled colony formation (Ray and Visick 2012). Further investigation revealed that deletion of *luxU* but not *luxO* leads to a similar delay, highlighting a branch within the signaling network that impacts biofilm development but not bioluminescence. In this current study, we report our discovery of another connection between the QS system and the *syp* locus, which expands our knowledge of the regulatory networks that *V. fischeri* has evolved to interact with its environment.

## Experimental Procedures

### Growth and media

*V. fischeri* strains were grown aerobically at 28°C in Luria-Bertani-Salt (LBS) broth (Graf et al. 1994) without supplemented glycerol. When necessary, chloramphenicol, tetracycline, and erythromycin were used at 2.5, 5.0, and 5.0 *μ*g mL^−1^, respectively. *Escherichia coli* strains were grown aerobically at 37°C in Luria-Bertani (LB) or brain–heart infusion (BHI) media. For fluorescence assay measurements, cells were resuspended in defined minimal medium (DMM): (50 mmol/L Tris-HCl [pH 7.5], 50 mmol/L MgSO_4_, 10 CaCl_2_, 300 mmol/L NaCl, 10 mmol/L KCl, 0.0058% K_2_HPO_4_, 10 *μ*mol/L FeSO_4_). TBSW (DeLoney-Marino et al. 2003) was used for motility assays.

### Strains and plasmids

*V. fischeri* strains and plasmids used in this study are listed in Table 1, and additional details of their construction are located in Supporting Information. All *V. fischeri* strains were derived from wild-type strain ES114 (Ruby et al. 2005; Mandel et al. 2008). *Escherichia coli* strains used in this work include EC100Dpir+ (Epicentre Biotechnologies, Madison, WI), TAM1 (Active Motif, Carlsbad, CA), *β*3914 (Le Roux et al. 2007), *π*3813 (Le Roux et al. 2007), CC118 *λpir* (Herrero et al. 1990), and GT115 (InvivoGen, San Diego, CA). Oligonucleotides used in this study are listed in Table S1 and were purchased from Integrated DNA Technologies, Inc. (Coralville, IA) (IDT).

**Table 1:** *Vibrio fischeri* strains and plasmids used in this study

Strain	Genotype	References
ES114	Wild-type *V. fischeri*	Boettcher and Ruby (1990); Ruby et al. (2005); Mandel et al. (2008)
CL59	*luxO*::*luxO*D47E	Lupp and Ruby (2005)
DRO1B3	*sypJ*::Tn*5*	This study
DRO216	*luxO*::*luxO*D47E *att*Tn*7*::*P*_*qrr1*_*-gfp erm*	This study
DRO222	*sypI*::Tn*5* [NT]	This study
DRO5F11	*sypI*::Tn*5*	This study
EVS102	Δ*luxCDABEG*	Bose et al. (2008)
KV4829	Δ*luxU*	This study
KV5069	Δ*sypL*	Shibata et al. (2012)
KV5972	Δ*luxQ*	This study
KV6010	Δ*luxP*	This study
KV6529	Δ*luxQ att*Tn*7*::*P*_*qrr1*_*-gfp erm*	This study
KV6530	Δ*luxU att*Tn*7*::*P*_*qrr1*_*-gfp erm*	This study
KV6549	Δ*luxP att*Tn*7*::*P*_*qrr1*_*-gfp erm*	This study
KV6629	Δ*sypL att*Tn*7*::*P*_*qrr1*_*-gfp erm*	This study
TIM303	*att*Tn*7*::*P*_*qrr1*_*-gfp erm*	This study
TIM305	Δ*qrr1*	Miyashiro et al. (2010)
TIM306	Δ*luxO*	Miyashiro et al. (2010)
TIM311	Δ*luxO att*Tn*7*::*P*_*qrr1*_*-gfp erm*	This study
TIM358	Δ*litR*	Miyashiro et al. (2010)
TIM374	Δ*luxPQ att*Tn*7*::*P*_*qrr1*_*-gfp erm*	This study
TIM394	Δ*sypK*	This study
TIM395	Δ*sypK att*Tn*7*::*P*_*qrr1*_*-gfp erm*	This study

Plasmid	Description	References
pCLD56	pKV282 *sypG*	Morris and Visick (2013)
pEVS107	R6Kori *oriT mini-*Tn*7 mob erm kan*	McCann et al. (2003)
pEVS79	pBC SK (+) *oriT cat*	Stabb and Ruby (2002)
pKV282	Mobilizable vector; TetR	Morris et al. (2011)
pLosTfoX	pEVS79 *tfoX*	Pollack-Berti et al. (2010)
pTM146	ColE1ori *bla cat kan gfp P*_*tetA*_*-mCherry*	Miyashiro et al. (2010)
pTM214	*lacI*^*q*^*P*_*trc*_*-mCherry*	Miyashiro et al. (2011)
pTM239	pEVS107 *P*_*qrr1*_*-gfp erm*	This study
pTM268	pVSV105 *P*_*qrr1*_*-gfp P*_*tetA*_*-mCherry*	Miyashiro et al. (2010)
pTM327	pEVS79 Δ*luxPQ*	This study
pTM367	pTM214 Δ*mCherry*::*sypK*	This study
pTM368	pTM214 Δ*mCherry*::*sypL*	This study
pTM375	pEVS79 Δ*sypK*	This study
pVAR18	Mobilizable suicide vector; Δ*luxU*	Ray and Visick (2012)
pVAR29	Mobilizable suicide vector; Δ*luxQ*	Ray and Visick (2012)
pVAR30	Mobilizable suicide vector; Δ*luxP*	Ray and Visick (2012)
pVAR48	pVSV105 *luxQ-FLAG*	This study
pVAR50	pVSV105 *luxQ*A216P-*FLAG*	This study
pVAR51	pVSV105 *luxQ*H378A-*FLAG*	This study
pVAR70	pKV282 *sypK-FLAG*	This study
pVSV105	R6Kori *ori*(pES213) RP4 *oriT cat*	Dunn et al. (2006)

### Transposon mutagenesis screen

The reporter plasmid pTM268 was introduced by conjugation into a Tn*5* transposon-mutant library of ES114 that has been previously described (Miyashiro et al. 2011). Recipients of the reporter plasmid were selected by plating the mating mixture onto LBS with 2.5 *μ*g mL^−1^ chloramphenicol. The resulting colonies were screened for elevated Green Fluorescent Protein (GFP) levels using a Leica MZFLIII fluorescence dissecting microscope (Leica Microsystems, Wetzlar, Germany), equipped with a GFP2 filter set.

To determine the transposon insertion site within each mutant, genomic DNA was extracted from 0.5 mL overnight LBS cultures using the MasterPure DNA Purification Kit (Epicentre Biotechnologies). Approximately 3-*μ*g genomic DNA was digested by EcoRI-HF (New England Biolabs, Ipswich, MA) in a 30-*μ*L reaction at 37°C. After 1 h at 37°C, EcoRI was heat inactivated at 65°C for 20 min. The enzyme was removed using the Wizard SV Gel and polymerase chain reaction (PCR) Clean-Up System (Promega, Madison, WI). The DNA was self-ligated using T4 DNA ligase (New England Biolabs), transformed by electroporation into EC100Dpir+ (Epicentre Biotechnologies), and selected on BHI containing 150 *μ*g mL^−1^ erythromycin. Plasmid DNA was extracted using the QIAprep Spin Miniprep Kit (Qiagen, Venlo, Netherlands) and sequenced at the UWBC DNA Sequencing Facility (University of Wisconsin-Madison) with transposon-specific primers pMJM10-Ext2 (CTAAAGAGGTCCCTAGCGATAAGC) and 170Ext (GCACTGAGAAGCCCTTAGAGCC).

### Fluorescence assay

Overnight LBS cultures containing 2.5 *μ*g mL^−1^ chloramphenicol were diluted 1:100 into fresh media and grown aerobically at 28°C. At OD_600_ ˜0.6, cultures were quickly cooled on ice. One-milliliter samples were spun at 15,000*g* for 5 min, and the pellets were resuspended in 350 *μ*L cold DMM. The OD_600_ and fluorescence of 100 *μ*L of each sample were determined in triplicate using a Tecan M1000 Pro Quadruple Monochromator Microplate Reader (Tecan Group, Mannedorf, Switzerland). For excitation and emission of GFP measurements, the monochromators were set to 488 ± 5 nm and 509 ± 5 nm, respectively. For excitation and emission of mCherry measurements, the monochromators were set to 587 ± 5 nm and 610 ± 5 nm, respectively. DMM was used as a blank for OD_600_ measurements. The fluorescence/OD_600_ was calculated by subtracting the autofluorescence levels associated with a nonfluorescent sample.

### Luminescence assay

Overnight LBS cultures were diluted 1:100 into fresh media and grown aerobically at 28°C. After 2 h, cultures were diluted 1:10 into media containing 3-oxo-C6 (Sigma, St. Louis, MO) at a final concentration of 120 nmol/L. At OD_600_ ˜0.6–0.8, a 100-*μ*L sample was sampled for luminescence using a GloMax 20/20 (Promega). Luminescence levels were normalized by the corresponding OD_600_ levels.

### Quantitative reverse transcription-PCR (qRT-PCR)

Overnight LBS cultures were diluted 1:100 into fresh media and grown aerobically at 28°C. At OD_600_ ˜0.5, cultures were quickly cooled on ice. To extract RNA, 1.5-mL samples were spun at 15,000*g* for 10 min, and the corresponding pellets were resuspended in 200 *μ*L QuickExtract RNA solution (Epicentre Biotechnologies). Samples were heated at 65°C with occasional mixing by vortexer. After 15 min, samples were cooled on ice. After 5 min, each sample was supplemented with 24-*μ*L DNase I buffer, 5 *μ*L Riboguard, and 10 *μ*L DNase I (Epicentre) and heated at 37°C. After 30 min, samples were cooled on ice. RNA was precipitated using 2× Tissue and Culture Solution and MPC Precipitation Reagent according to manufacturer's instructions. A second round of DNase I treatment was performed by resuspending RNA in 94 *μ*L DNase I 1x buffer, 2 *μ*L Riboguard, and 4 *μ*L DNase I. After 1 h at 37°C, RNA was precipitated as described above. RNA was resuspended in 15 *μ*L of nuclease-free water (IDT). The concentration of RNA was measured using a Nanodrop (Thermo Scientific, Waltham, MA).

RT reactions were performed starting from 4 *μ*g of total RNA, using AMV Reverse Transcriptase (Promega, Madison, WI) and Random Primers (Promega), according to the manufacturer's instructions. Negative controls were performed in the same manner but without AMV Reverse Transcriptase. The resulting cDNA samples were diluted 1:80 in nuclease-free water. Each 25-*μ*L reaction mixture for qRT-PCR consisted of 10-*μ*L cDNA, iQ SYBR Green Supermix diluted to 1×, and 500 nmol/L of each primer. qRT-PCR was performed in an iCycler Thermal Cycler (Bio-Rad Laboratories, Hercules, CA) with two technical replicates/biological replicate as follows: 3 min at 95°C, 40 times (15 sec at 95°C, 30 sec at 60°C, 30 sec at 72°C), 1 min at 60°C. A melting curve was recorded at the end of the PCR amplification (from 60°C to 100°C) to confirm that a unique transcript product had been amplified. To calculate PCR efficiencies, standard curves were plotted using five 10-fold dilutions of a mixture containing 2.5 *μ*L of each cDNA reaction diluted 1:2 with nuclease-free water. Primer sets exhibited amplification efficiencies (E) of 1.94–2.03. Gene expression values were calculated using the E^−Ct^ method, where Ct corresponds to the threshold cycle. Expression levels of each gene were normalized by the corresponding wild-type expression level. For each gene, comparison between strains was performed using an unpaired *t*-test with *P*-values adjusted using false discovery rate correction (Prism, v. 6.03, La Jolla, CA).

### Motility assay

Overnight LBS cultures supplemented with 2.5 *μ*g mL^−1^ chloramphenicol were diluted 1:100 into LBS supplemented with chloramphenicol and 100 *μ*mol/L IPTG. Cultures were standardized to OD = 0.2 and inoculated into Tryptone-based Seawater (TBSW) motility plates containing chloramphenicol and Isopropyl Beta-D-1-thiogalactopyranoside (IPTG). Assays were performed as described previously (DeLoney-Marino et al. 2003).

### Western blotting

Western blot analysis was used to analyze the levels of epitope (FLAG)-tagged *luxQ* from KV6529 containing pVAR48 (wild-type *luxQ*-FLAG), pVAR50 (*luxQ-A216P-FLAG*), or pVAR51 (*luxQ-H378A-FLAG*) and either the vector control (pKV282) or the *sypK* overexpression plasmid pVAR70. Briefly, cultures were grown overnight with shaking in LBS containing tetracycline and chloramphenicol. Samples were collected and standardized to an OD_600_ = 3.5, resuspended in 500-*μ*L 2x SDS-loading buffer (4% SDS, 10% 2-mercaptoethanol, 0.005% bromophenol blue, 20% glycerol, 0.1 mol/L Tris pH 7), boiled for 5 min, and then loaded onto a 10% SDS polyacrylamide gel. After electrophoresis, proteins were transferred to a polyvinylidene fluoride membrane (PVDF) and probed with an anti-FLAG antibody (Sigma-Aldrich, St. Louis, MO). Protein bands were visualized using a horseradish peroxidase-conjugated secondary antibody and ECL reagents (Pierce Biotechnology, Rockford, IL).

## Results

### Identification of *syp* genes that affect *qrr1* expression

We have previously shown that the LuxU-LuxO phosphorelay activates *qrr1* to control the level of the transcription factor LitR (Miyashiro et al. 2010). To further characterize this branch within the QS network of *V. fischeri*, we initiated a genetic screen by introducing the *qrr1* transcriptional reporter plasmid pTM268 into a Tn*5* transposon-mutant library of the wild-type *V. fischeri* strain ES114. The plasmid pTM268 contains the *qrr1* promoter cloned upstream of *gfp*, and the constitutively expressed *tetA* promoter cloned upstream of *mCherry*. The GFP/mCherry fluorescence ratio of cells harboring pTM268 provides a quantitative measure of *qrr1* expression. In colonies of wild-type *V. fischeri* harboring pTM268, the level of GFP fluorescence is low (data not shown), presumably due to the high cell density conditions within colonies repressing *qrr1* expression.

**Figure 2:**
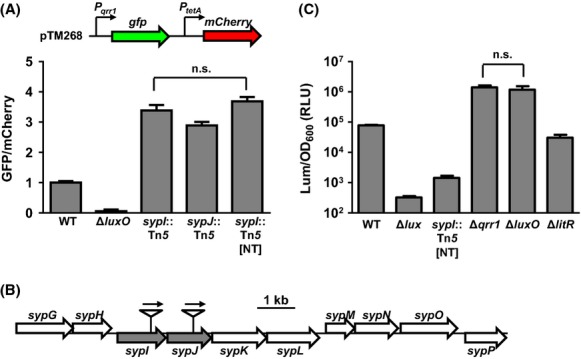
Mutants with a transposon insertion in the *sypIJKL* operon have enhanced *qrr1* expression. (A) Levels of *qrr1* expression in WT (ES114), Δ*luxO* (TIM306), *sypI*::Tn*5* (DRO5F11), *sypJ*::Tn*5* (DRO1B3), and *sypI*::Tn*5* [NT] (DRO222) harboring the reporter plasmid pTM268. The nonfluorescent strain ES114 harboring pVSV105 was used to calculate cellular levels of GFP and mCherry. Graphical and error bars represent the averages and standard deviations of triplicate biological replicates, respectively. One-way ANOVA with Tukey's multiple comparisons test show significance (*P*-value <0.01) between columns, except for the comparison labeled not significant (n.s.). Experiment was performed three times, with similar results. (B) Transposon insertion sites of mutants examined in (A). Genes disrupted by a transposon are highlighted in gray. Arrows above transposon insertions indicate direction of *erm* gene transcription. (C) Luminescence levels of WT (ES114), Δ*lux* (EVS102), *sypI*::Tn*5* [NT] (DRO222), Δ*qrr1* (TIM305), Δ*luxO* (TIM306), and Δ*litR* (TIM358) in response to 120 nmol/L 3-oxo-C6. Graphical and error bars represent the averages and standard deviations of triplicate biological replicates, respectively. One-way ANOVA with Tukey's multiple comparisons test on log-transformed data show significance (*P*-value <0.01) between columns, except for the comparison labeled not significant (n.s.). Experiment was performed three times, with similar results.

We screened over 100,000 colonies and isolated ˜100 clones with elevated levels of GFP. In this study, we report our characterization of two mutants isolated from this genetic screen. Both mutants displayed *qrr1* expression levels that were approximately threefold higher than wild-type cells (Fig. 2A). Sequencing of the transposon insertion site in each mutant revealed an insertion in either *VF_A1028* (*sypI*) or *VF_A1029* (*sypJ*) (Fig. 2B). To determine whether the phenotype of elevated *qrr1* expression was linked to the transposon, we reintroduced the *sypI* transposon insertion into ES114 by transformation. The resulting strain, DRO222 (designated *sypI*::Tn*5* [NT]), showed *qrr1* expression levels comparable to the original transposon insertion mutant (*sypI*::Tn*5*), indicating that the transposon insertion in *sypI* is linked to elevated *qrr1* expression (Fig. 2A). Hereafter, the *sypI*::Tn*5* [NT] mutant is termed the *sypI* mutant.

LitR indirectly enhances luminescence in *V. fischeri* by binding the intergenic region between *luxR* and *luxI* to positively regulate *luxR* expression (Fidopiastis et al. 2002; Miyashiro et al. 2010). Because Qrr1 posttranscriptionally represses *litR*, cells expressing *qrr1* are predicted to exhibit low luminescence levels. Relative to wild-type cells, Δ*qrr1* and Δ*luxO* mutants become 18- and 15-fold brighter, respectively, and a Δ*litR* mutant is 2.5-fold dimmer (Fig. 2C). Consistent with high levels of *qrr1* expression, the *sypI* mutant is 55-fold dimmer than wild-type cells. Together, these results suggest that the *syp* locus can affect *qrr1* expression and QS phenotypes in *V. fischeri*.

### Polar effect of transposon insertion on *syp* expression

Both *sypI* and *sypJ* are predicted to encode glycosyltransferases, and the effects of their disruption on *syp*-mediated biofilm formation have recently been determined (Shibata et al. 2012). Whereas *sypJ* is required for biofilm formation, a deletion of *sypI* only delays biofilm formation. We were unable to formulate a simple model that could account for the ability of the two different glycosyltransferases to affect *qrr1* expression. However, in each transposon mutant, the promoter associated with the erythromycin resistance marker (*erm*) was oriented in the same direction as the *syp* locus (Fig. 2B). In addition, the insertions, which were within different genes in the same operon, resulted in similarly high levels of *qrr1* expression (Fig. 2A). Therefore, we hypothesized that the elevated level of *qrr1* expression detected in each mutant was due to the activation of genes downstream of the transposon insertion rather than to the disruption of either *sypI* or *sypJ*.

**Figure 3:**
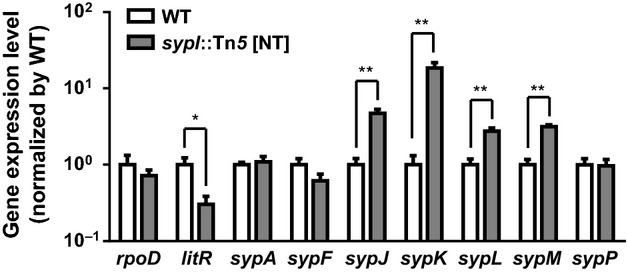
Polar effects of transposon insertion on *syp* gene expression. Quantitative reverse-transcriptase PCR analysis of various genes in the *sypI* mutant (DRO222) relative to WT (ES114). Genes tested are *VF_2254* (*rpoD*), *VF_2177* (*litR*), *VF_A1020* (*sypA*), *VF_A1025* (*sypF*), *VF_A1029* (*sypJ*), *VF_A1030* (*sypK*), *VF_A1031* (*sypL*), *VF_A1032* (*sypM*), and *VF_A1035* (*sypP*). Values are of quadruplicate biological replicates and normalized by wild-type levels. Error bars indicate ± 1 SD. Comparisons with significance based on unpaired *t*-tests are shown with *(*P*-value <0.01) and **(*P*-value <0.001), where *P*-values are adjusted using false discovery rate correction.

To test this hypothesis, we examined the transcript levels of various genes in the *sypI* mutant. We found that *litR* levels were reduced 3.3-fold in the *sypI* mutant relative to wild-type cells (Fig. 3), consistent with the elevated expression of *qrr1* in the mutant. We also found that the *sypI* mutant exhibited higher transcription levels of *sypJ* (4.7-fold), *sypK* (18.5-fold), and *sypL* (2.8-fold). Because our original screen also identified a *sypJ* mutant, we conclude that the elevated level of *qrr1* expression in the *sypI* mutant is independent of *sypJ*. To determine whether the transposon insertion resulted in a general activation of the *syp* locus, we also examined several other *syp* genes. While the level of *sypM*, which is contained within the adjacent operon (Fig. 2B), was elevated 3.2-fold (Fig. 3), three other genes (*sypA*, *sypF*, and *sypP*) displayed wild-type levels of transcription in the *sypI* mutant. These results demonstrate that the transposon insertion leads to the increased expression of several downstream genes, but not of the entire locus.

### Induction of *sypK* results in *qrr1* expression

**Figure 4:**
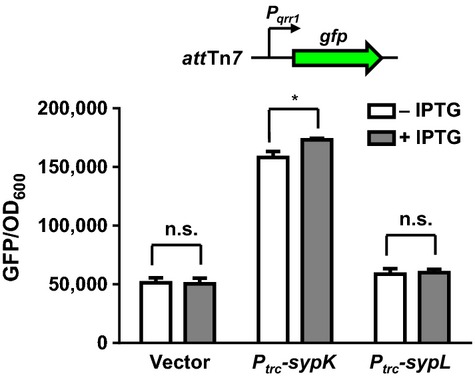
Overexpression of *sypK* activates *qrr1* expression. Levels of *qrr1* expression in the reporter strain TIM303 harboring vector (pTM214), *P*_*trc*_*-sypK* (pTM367), or *P*_*trc*_*-sypL* (pTM368) grown ±100 *μ*mol/L IPTG. The wild-type strain ES114 harboring pTM214 was used as the GFP-negative control for quantifying GFP levels. Graphical and error bars represent the averages and standard deviations of triplicate biological replicates, respectively. Comparisons with significance based on two-way ANOVA with Tukey's multiple comparisons are shown with *(*P*-value <0.01). Comparisons between pTM367-harboring strains with strains harboring either pTM214 or pTM368 are significant (*P-*value <0.001). Experiment was performed three times, with similar results.

To determine whether increased expression of *sypK* or *sypL* leads to *qrr1* expression, we separately cloned *sypK* and *sypL* on a plasmid downstream of the IPTG-inducible *trc* promoter. For experiments involving overexpression vectors, we monitored *qrr1* expression using a GFP transcriptional reporter integrated into the chromosome at the Tn*7* insertion site. We found that basal expression of *sypK* from the *trc* promoter was sufficient to increase *qrr1* levels at least threefold higher than wild-type levels (Fig. 4). Addition of IPTG to the growth medium resulted in a slight but significant increase in *qrr1* expression. In contrast, we did not detect any change in *qrr1* expression when using the IPTG-inducible *sypL* construct. These results indicate that overexpression of *sypK* is sufficient to activate *qrr1* expression.

**Figure 5:**
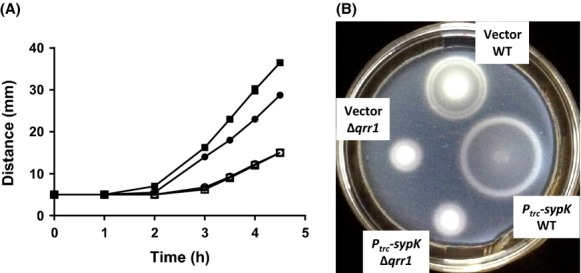
Overexpression of *sypK* enhances *qrr1*-dependent motility. (A) Soft-agar motility migration distances by WT (ES114; closed symbols) and Δ*qrr1* (TIM305; open symbols) harboring vector (pTM214; circles) or *P*_*trc*_*-sypK* (pTM367; squares). TBSW motility plates contained chloramphenicol and 100 *μ*mol/L IPTG. Points and error bars (too small to visualize) represent means and standard deviations of spot diameter (in mm) for quadruplicate biological replicates, respectively. Experiment was performed twice, with similar results. (B) Image of motility plate used in (A) at 4 h.

Previous studies have shown that *V. fischeri* also uses the LuxU-LuxO QS pathway to regulate motility (Lupp and Ruby 2005; Cao et al. 2012). In particular, *V. fischeri* cells become hypermotile in the absence of *litR*. To test whether SypK has an effect on motility, we examined on soft agar the motility of cells expressing *sypK*. Compared to wild-type motility levels, cells with induced *sypK* expression were hypermotile (Fig. 5A and B). Furthermore, this hypermotility phenotype depended on Qrr1, as shown by the overall reduced motility associated with mutants containing the Δ*qrr1* allele. Together, these results demonstrate that expression of *sypK* leads to higher motility due to increased *qrr1* transcription.

### SypK modulates LuxO phosphorylation via LuxQ

The *sypK* gene is predicted to encode an oligosaccharide translocase. SypK is required for *V. fischeri* to form *syp*-mediated biofilms as well as to colonize *E. scolopes* juvenile squid (Shibata et al. 2012). In addition, SypK also contributes to the production of outer membrane vesicles under biofilm-inducing conditions (Shibata and Visick 2012). We concluded that, because of its predicted function as an oligosaccharide translocase, SypK is unlikely to directly regulate *qrr1* expression. Therefore, we assayed *qrr1* expression in response to *sypK* induction in various QS mutants of *V. fischeri* to determine whether, and at what step, SypK interacts with the known QS pathway.

**Figure 6:**
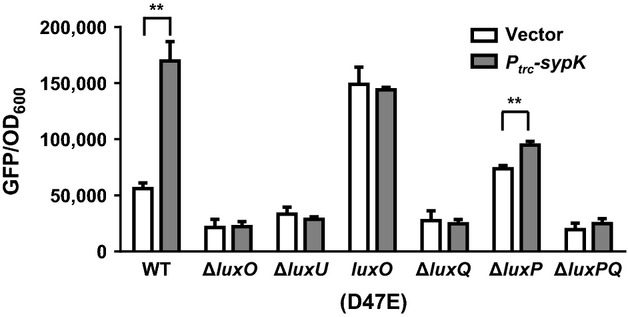
SypK modulates the LuxU-LuxO phosphorelay via LuxQ. Levels of *qrr1* expression in WT (TIM303), Δ*luxO* (TIM311), Δ*luxU* (KV6530), *luxO*D47E (DRO216), Δ*luxQ* (KV6529), Δ*luxP* (KV6549), Δ*luxPQ* (TIM374) harboring vector (pTM214) or *P*_*trc*_*-sypK* (pTM367) grown in the presence of 100 *μ*mol/L IPTG. The wild-type strain ES114 harboring pTM214 was used as the GFP-negative control for quantifying GFP levels. Graphical and error bars represent the averages and standard deviations of triplicate biological replicates, respectively. Comparisons with significance based on unpaired *t*-tests are shown with **(*P*-value <0.001), where *P*-values are adjusted using false discovery rate correction. Experiment was performed three times, with similar results.

We first examined the response of *qrr1* expression to *sypK* induction in the absence of either LuxO or LuxU, which are the phosphorelay proteins that directly control *qrr1* expression (Fig. 1). We found that *qrr1* expression remained at or lower than the wild-type level in either Δ*luxO* or Δ*luxU* mutants, regardless of whether *sypK* was induced (Fig. 6), indicating that both proteins are required for SypK-dependent activation of *qrr1*. We next examined *qrr1* expression in a strain containing a *luxO*D47E allele, which expresses a variant of LuxO that mimics the phosphorylated state of LuxO (Lupp and Ruby 2005). We found that the level of *qrr1* expression in the *luxO*D47E mutant was higher than in wild-type cells (Fig. 6). Importantly, *qrr1* expression in the *luxO*D47E mutant was independent of *sypK* induction, suggesting that an intact LuxU-LuxO phosphorelay is required for SypK to increase *qrr1* expression (Fig. 6). Together, these results indicate that SypK acts upstream of the LuxU-LuxO phosphorelay to affect *qrr1* expression.

Because LuxO phosphorylation is controlled by upstream kinases, we next examined the effect of the signaling proteins controlling the LuxU-LuxO phosphorelay. Specifically, we assessed whether SypK activation of the LuxU-LuxO phosphorelay depends on the sensor kinase LuxQ or the AI-2 receptor protein LuxP, which has been shown in *V. harveyi* and *V. cholerae* to control the kinase and phosphatase activities of LuxQ (Neiditch et al. 2005, 2006; Shikuma et al. 2009). Induction of *sypK* in either *ΔluxQ* or Δ*luxPQ* mutants did not result in *qrr1* expression (Fig. 6), indicating that SypK-mediated activation of *qrr1* requires LuxQ. Relative to wild-type cells, deletion of *luxP* resulted in increased *qrr1* expression (Fig. 6), which is consistent with the inhibitory function LuxP exhibits toward the kinase activity of LuxQ in *V. harveyi* (Neiditch et al. 2005). We found a slight but significant increase in *qrr1* expression when *sypK* was induced in the Δ*luxP* mutant, suggesting that the ability of SypK to activate LuxQ may also involve LuxP. Taken together, our results suggest that SypK activates *qrr1* by stimulating the LuxP/Q complex, which modulates the LuxU-LuxO phosphorelay.

**Figure 7:**
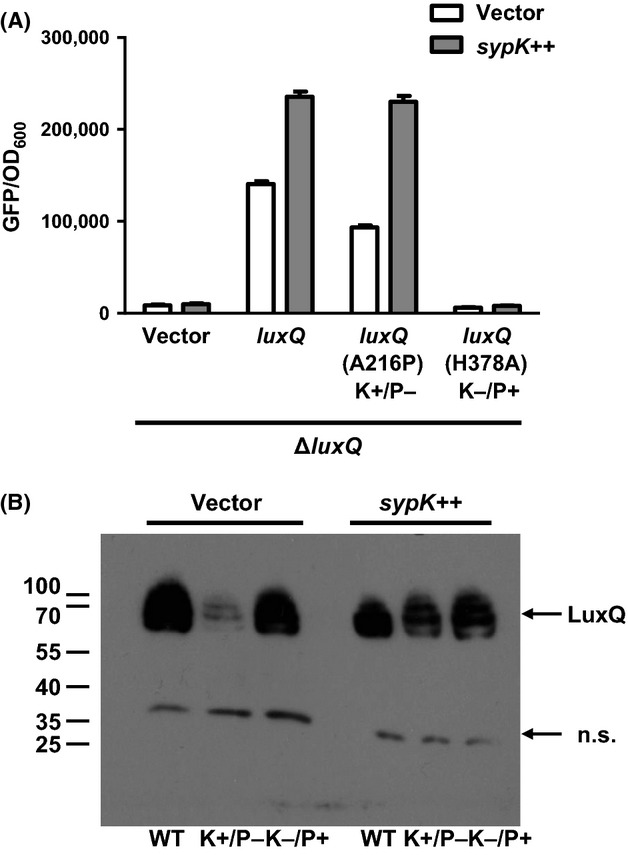
Kinase activity of LuxQ is required for SypK activation of *qrr1* expression. (A) Levels of *qrr1* expression in Δ*luxQ* strain KV6529 harboring a *luxQ-FLAG* variant plasmid (vector [pVSV105], *luxQ* [pVAR48], *luxQ* [A216P] K+/P− [pVAR50], or *luxQ* [H378A] K−/P+ [pVAR51]) and a *sypK* plasmid (vector [pKV282] or *sypK*++ [pVAR70]). The wild-type strain ES114 harboring pVSV105 and pKV282 was used as the nonfluorescent control for quantifying fluorescence levels. The copy of *sypK* in plasmid pVAR70 contains the *FLAG* tag. Graphical and error bars represent the averages and standard deviations of triplicate biological replicates, respectively. Experiment was performed three times, with similar results. (B) Western blot of *ΔluxQ* strain KV6529 harboring a *luxQ-FLAG* variant plasmid (WT [pVAR48], K+/P− [pVAR50], or K−/P+ [pVAR51]) and a *sypK* plasmid (vector [pKV282] or *sypK++* [pVAR70]). Anti-FLAG antibodies were used to detect the variants of LuxQ-FLAG. Numbers to the left indicate molecular-weight marker positions (in kDa). A nonspecific band detected throughout the samples is designated as “n.s.”. A band corresponding to SypK-FLAG was not detected in this experiment. Experiment was performed twice, with similar results.

Like many histidine kinases, LuxQ is bifunctional, with both kinase and phosphatase activities (Freeman and Bassler 1999a, 1999b). To determine which enzymatic activity of LuxQ is modulated by SypK, we used two LuxQ variants, A216P or H378A, that exhibit only kinase (K+/P−) or phosphatase (K−/P+) activities, respectively (Neiditch et al. 2006; Ray and Visick 2012). Consistent with the results shown in Figure 6, we found that *qrr1* expression remained low in a Δ*luxQ* mutant, regardless of whether *sypK* was overexpressed (Fig. 7A). Introduction of *luxQ* into the Δ*luxQ* mutant in trans resulted in an increased expression of *qrr1*. The level of *qrr1* expression in this strain was even higher than in wild-type cells (Fig. 6), presumably due to the overexpression of *luxQ* from the multicopy plasmid pVAR48. In *V. cholerae*, a similar activation of the analogous LuxU-LuxO phosphorelay by overexpression of the *luxQ* homologue has been observed (Shikuma et al. 2009). Similar to the results shown in Figure 6, the overexpression of *sypK* in *V. fischeri* harboring *luxQ* in trans also led to the increased *qrr1* expression (Fig. 7A). In the presence of the K+/P− mutation *luxQ*A216P, *qrr1* was expressed, albeit at lower levels than in the presence of *luxQ*. This lower level of *qrr1* expression may be attributed to the overall lower levels of the LuxQA216P variant relative to wild-type LuxQ (Fig. 7B). Overexpression of *sypK* in this background resulted in a high level of *qrr1* expression that was comparable to the level induced by *sypK* in cells expressing wild-type LuxQ (Fig. 7A). Finally, the expression of *qrr1* was low in cells harboring the K−/P+ variant of LuxQ regardless of *sypK* induction. As shown in Figure 7B, the LuxQH378A variant was produced at levels comparable to wild-type LuxQ. Taken together, these data suggest that the kinase activity of LuxQ is required for SypK-dependent regulation of *qrr1* expression.

### Induction of the *syp* locus results in *qrr1* expression

Our results indicate that overexpression of *sypK* is sufficient to activate *qrr1* expression in wild-type cells. Therefore, we hypothesized that conditions that activate transcription of the *syp* locus would also lead to increased *qrr1* expression in a *sypK*-dependent manner. Because the formation of *syp*-mediated biofilms is linked to SypK, and cellular physiology significantly varies between planktonic and biofilm states, we sought conditions that would activate transcription of the *syp* locus but prevent biofilm formation. Previous work has demonstrated that in trans expression of *sypG*, which encodes a transcription factor, results in transcription of the *syp* locus without concomitant biofilm formation (Yip et al. 2005; Hussa et al. 2008). Further analysis of this condition has determined that the lack of biofilm formation is due to an inhibitory activity by the regulator SypE (Morris and Visick 2013).

**Figure 8:**
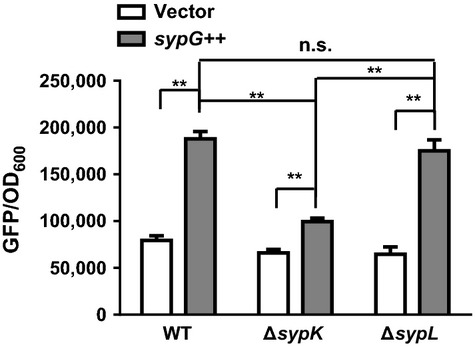
Expression of *qrr1* increases with SypG-mediated activation of *syp* locus. Levels of *qrr1* expression in WT (TIM303), Δ*sypK* (TIM395), and Δ*sypL* (KV6629) harboring pKV282 (vector) or pCLD56 (*sypG++*). The wild-type strain ES114 harboring pCLD56 was used as the nonfluorescent control for quantifying fluorescence levels. Graphical and error bars represent average and standard deviation of three biological replicates, respectively, from a representative experiment performed twice. Comparisons with significance based on two-way ANOVA with Tukey's multiple comparisons test shows significance **(*P*-value <0.001) between columns, except for the comparison labeled nonsignificant (n.s.) and for comparisons between columns for vector controls, which are also nonsignificant.

We thus assayed *qrr1* expression in cells harboring either a multicopy plasmid containing *sypG* (pCLD56) or a vector control (pKV282). Wild-type cells that overexpressed *sypG* exhibited ˜2.4-fold higher *qrr1* expression levels than did vector-containing cells (Fig. 8). This level of *qrr1* activity was comparable to that in cells overexpressing *sypK* (Fig. 4). The level of *qrr1* activation in response to *sypG* decreased to 1.5-fold in the absence of *sypK* (Fig. 8). In addition, the 2.7-fold *qrr1* activation by *sypG* in a Δ*sypL* mutant was comparable to the response in wild-type cells, confirming our results that showed *qrr1* expression is independent of *sypL* induction (Fig. 4). Thus, our results show that conditions that lead to transcription of the *syp* locus, that is, biofilm formation, also activate *qrr1* expression.

## Discussion

We initiated this study to identify novel regulators of *qrr1*, which encodes a sRNA conserved among members of the *Vibrionaceae* family (Lenz et al. 2004; Miyashiro et al. 2010; Weber et al. 2011; Shao and Bassler 2012). Our results show that the putative oligosaccharide translocase SypK can interact with the QS network to control *qrr1* levels in *V. fischeri*. In particular, our data are consistent with the conclusion that SypK exerts its impact at or above the LuxP/Q complex in a manner that depends on the kinase activity of LuxQ (Figs. 6, 7). Because SypL, which is another predicted inner membrane protein, does not activate *qrr1* expression (Fig. 4), we posit that the increase in LuxQ signaling by SypK is not due to a general perturbation of the protein composition within the inner membrane. The activation of the LuxU-LuxO phosphorelay resulting from SypK overexpression, either as an individual protein or in the context of activation of the *syp* locus (Fig. 8), leads to the expression of *qrr1*. Finally, our results show that the interaction between SypK and the QS pathway is sufficient to affect cellular behaviors associated with quorum sensing in *V. fischeri*, for example, bioluminescence and motility (Figs. 2C, 5).

**Figure 9:**
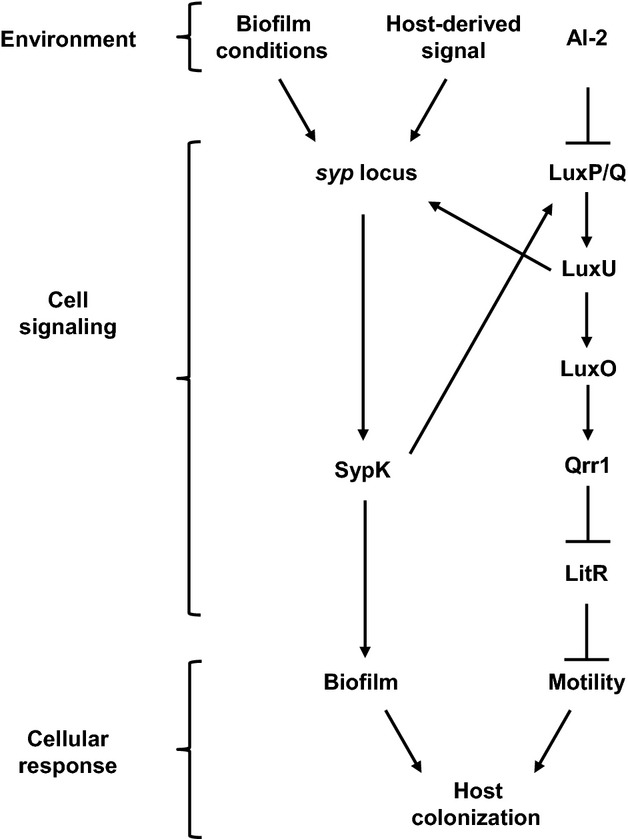
Model of integrated biofilm and QS regulatory networks in *Vibrio fischeri*. Environmental cues, including conditions associated with the host during initial colonization of the squid light organ, activate the *syp* locus. In addition to participating in biofilm formation, SypK activates the LuxU-LuxO phosphorelay via the LuxP/Q complex. The resulting expression of *qrr1* leads to enhanced flagellar-based motility, which may contribute to host colonization (see Discussion).

From these results, we have generated the model presented in Figure 9. Under conditions that result in transcription of the *syp* locus, the *sypK* gene will be expressed, leading to the formation of this putative oligosaccharide translocase within the inner membrane. While contributing to biofilm formation, SypK can also interact with the QS pathway via LuxQ. Our results in Figure 7B suggest that the levels of the K+/P− variant of LuxQ may be influenced by SypK. One interpretation of these data consistent with our other results is that SypK stabilizes the kinase form of LuxQ; however, a clear understanding of this pathway awaits a more complete study. Regardless, the net result of *sypK* induction is activation of the LuxU-LuxO phosphorelay and, consequently, *qrr1* expression. Whether the resulting increase in LuxU phosphorylation can further enhance *syp* transcription via SypG (Ray and Visick 2012) remains unknown; further characterization of the signaling pathway directly controlling the *syp* locus is required to determine whether a potential positive feedback loop is present. Interestingly, this regulatory link between SypK and LuxQ suggests a mechanism that enables *V. fischeri* cells densely packed within a biofilm to activate the LuxU-LuxO phosphorelay even in the presence of AI-2, which is an autoinducer broadly used for bacterial QS.

The threefold increase in *qrr1* expression observed in the transposon insertion mutants resulted in a 55-fold decrease in luminescence (Fig. 2A and C). Such dramatic effects on cellular luminescence from small changes in the expression of genes involved in QS have been previously reported. For instance, deletion of *arcA*, which encodes a response regulator that responds to redox conditions, results in a 500-fold effect on luminescence despite only changing *luxI* transcription by 10-fold (Bose et al. 2007; Septer and Stabb 2012). Similarly, deletion of *litR* results in less than a twofold effect on *luxR* expression, but decreases luminescence by 10-fold (Miyashiro et al. 2010). The results presented here provide another example of the exquisite sensitivity of bioluminescence to QS.

We also found that the *sypI* mutant is 21-fold dimmer than a Δ*litR* mutant (Fig. 2C). Because Qrr1 represses *litR* mRNA levels, we anticipated that the luminescence levels of strains overexpressing *qrr1* would be higher than the Δ*litR* mutant. Our surprising result with the *sypI* mutant may be due, in part, to an indirect effect from overexpressed Qrr1 titrating free Hfq, a condition that occurs in *E. coli* when an sRNA is overexpressed (Moon and Gottesman 2011). In *V. fischeri*, lower levels of free Hfq may result in inefficient posttranscriptional regulation by other sRNAs involved in regulating luminescence.

We found that overexpression of SypG also enhances *qrr1* expression in a SypK-independent manner (Fig. 8). A recent study has revealed that the SypG regulon extends beyond the *syp* locus (Ray et al. 2013), and several of these genes appear to impact bioluminescence (V. A. Ray and K. L. Visick, unpubl. results). Future studies will determine how these SypG-regulated genes affect bioluminescence and whether the mechanism involves *qrr1*.

All sequenced *Vibrionaceae* members possess a *luxQ* homologue within their genomes; however, the presence of *sypK* and the remaining *syp* genes appears to be species dependent. In addition to *V. fischeri*, the pathogens *Vibrio parahaemolyticus* and *Vibrio vulnificus* have been reported to each contain the *syp* locus, including a *sypK* homologue (Yip et al. 2005). Homologues of *sypK* are also present within the genomes of *Aliivibrio salmonicida* (*VSAL_II0302*), *V. harveyi* (*VIBHAR_02224*), *Photobacterium profundum* (*PBPRA1735*), and *Vibrio splendidus* (*VS_2150*). Notably, the *syp* locus is absent from the genomes of *V. cholerae*, *Vibrio anguillarum*, and *Vibrio furnissii*. As future studies uncover the mechanism underlying the interaction between SypK and LuxQ, they will also provide opportunities to determine how *sypK* and, more generally, the *syp* locus have coevolved with the core QS network of the *Vibrionaceae*, and whether the regulatory link is conserved.

Our finding that a protein involved in biofilm formation can also function in signaling through the QS pathway inverts the traditional view of the role of bacterial QS during biofilm development. Generally, many genes that are involved in forming biofilms are regulated by QS systems. For example, in *V. cholerae*, expression of the *vps* exopolysaccharide gene cluster is downregulated by HapR, the LitR homologue in *V. cholerae*, (Hammer and Bassler 2003; Zhu and Mekalanos 2003). In *V. vulnificus*, the LitR homologue SmcR was recently shown to control transcription of the capsular polysaccharide (CPS) gene cluster (Lee et al. 2013). From this latter work, it has been proposed that QS within mature biofilms results in the production of cell-associated CPS, which decreases the hydrophobicity of the cell surface. As a result, *V. vulnificus* cells are released from biofilms with high cell density, thereby providing a mechanism to control the overall size of a biofilm. QS control of biofilm formation has also been observed in non-*Vibrionaceae* bacteria, including the pathogen *Pseudomonas aeruginosa*, which uses hierarchically arranged LasR/LasI and RhlR/RhlI QS systems to control biofilm development, in addition to virulence, motility, and antibiotic resistance (Williams and Camara 2009). The study we report here demonstrates that induction of a gene involved in biofilm development is able to influence QS. Whether this regulatory link represents a general phenomenon in bacteria or is instead specific to *V. fischeri* remains unknown.

Is the regulatory link between SypK and the QS network relevant to the known biology of *V. fischeri*? Because biofilm formation is often correlated with a sessile, community lifestyle, it seems somewhat counterintuitive for microbes to activate a signaling cascade associated with the planktonic, that is, low cell density, state while actively developing a biofilm. However, the general developmental cycle of a biofilm includes dispersal, which describes the stage when a subset of cells leaves the matrix to initiate biofilm formation on another surface (McDougald et al. 2012). In *V. cholerae*, mutation of *luxO* represses biofilm formation, reduces motility, and promotes cellular detachment from biofilms (Zhu et al. 2002; Zhu and Mekalanos 2003). Consequently, inactivation of the LuxU-LuxO phosphorelay via QS can prime cells within mature, densely packed biofilms for dispersal. In *V. fischeri*, the effect on motility by the LuxU-LuxO phosphorelay is similar to that observed in *V. cholerae*: mutation of either *luxO* or *qrr1* results in attenuated motility (Lupp and Ruby 2005) (Fig. 5). Therefore, the increased motility from SypK-dependent *qrr1* activation may enhance *V. fischeri* dispersal from *syp*-mediated biofilms.

Activation of *qrr1* expression by SypK may also play a role during the initiation of the squid-*Vibrio* symbiosis. The current model of initial host colonization is a two-step process, in which *V. fischeri* cells first attach individually to host cilia and then aggregate in a *syp*-dependent manner outside the light-organ pores (Altura et al. 2013). The *syp* genes, including *sypK*, are required for *V. fischeri* to efficiently colonize juvenile squid (Yip et al. 2005; Shibata et al. 2012). Our model predicts that activation of the *syp* locus will result in high *qrr1* expression (Fig. 9), which has been shown to repress *litR* mRNA levels (Miyashiro et al. 2010). *V. fischeri* cells containing a *litR* deletion allele are able to outcompete wild-type cells in host colonization (Fidopiastis et al. 2002; Miyashiro et al. 2010). We hypothesize that by linking SypK and the QS network, *V. fischeri* symbionts can escape via flagellar-based motility from the exopolysaccharide matrix secreted during the aggregation stage. The residual biofilm may hinder other cells from entering the light organ, thereby contributing to the winnowing process during the initial establishment of symbiosis (Nyholm and McFall-Ngai 2004). Future molecular-based studies will help resolve the interconnectivity of SypK in biofilm formation, host colonization, and quorum sensing in *V. fischeri*.

## Supporting Information

Table S1 Primers used in this study.
