# Supplementary material for: The putative oligosaccharide translocase SypK connects biofilm formation with quorum signaling in Vibrio fischeri
Source: Microbiologyopen. 2014 Sep 25;3(6):836–48. doi: 10.1002/mbo3.199 (PMC4263508; doi:10.1002/mbo3.199)
Supplement: Table S1 — Primers used in this study. [file mbo30003-0836-sd1.pdf]

Supporting Information

The putative oligosaccharide translocase SypK connects biofilm formation with quorum signaling in *Vibrio fischeri*

Tim Miyashiro[1,2,*], Dane Oehlert[2], Valerie A. Ray[3], Karen L. Visick[3], and Edward G. Ruby[2]

[1] Department of Biochemistry and Molecular Biology
Eberly College of Science
The Pennsylvania State University
University Park, PA 16802, USA

[2] Department of Medical Microbiology and Immunology
University of Wisconsin School of Medicine and Public Health
Madison, WI 53706, USA

[3] Department of Microbiology and Immunology
Loyola University Medical Center
Maywood, IL 60153, USA

* Corresponding author
Email: tim14@psu.edu
Phone: (+1) 814 865 1916
Fax: (+1) 814 863 7024

This file contains:

Strain Construction
Primer List (Table S1)
References

Strain Construction

Natural transformation to generate DRO222. Template DNA for natural transformation was prepared from the plasmid generated for sequencing the transposon insertion site within DRO5F11. Approximately 1 µg plasmid DNA was digested with EcoRI-HF (New England Biolabs, Ipswich, MA, USA) in a 25-µl reaction at 37°C. After 1 h, the reaction was stopped by using a QIAquick PCR Purification Kit (Qiagen, Venlo, Netherlands) according to the manufacturer's instructions and eluted with 40 µl water.

A culture of ES114 harboring pLosTfoX was grown overnight at 28°C with aeration in DMM containing 9.9 mM glucosamine and 2.5 µg/ml chloramphenicol. The culture was diluted 1:4 into fresh media and grown at 28°C. After 1.5 h, 500 µl culture was combined with DNA template described above and incubated at room temperature. After 30 min, 1 ml LBS was added to the mixture and grown at 28°C overnight with aeration. The culture was concentrated by centrifugation and the resulting pellet was resuspended in 150 µl LBS, which was spread onto LBS agar containing 5.0 µg/ml erythromycin and incubated at 28°C. An erythromycin-resistant colony was selected as DRO222 and further characterized as described in the main text.

Allelic exchange vectors. To construct the plasmid pTM327, which contains the Δ*luxPQ* allele that removes the codons corresponding to amino acids 5-372 of LuxP and 1-734 of LuxQ, the regions surrounding *luxPQ* were amplified from ES114 genomic DNA by PCR using primers luxPQ-5-XhoI-U/luxPQ-5-BamHI-L and luxPQ-3-BamHI-U/luxPQ-3-XbaI-L, digested by XhoI/BamHI and BamHI/XbaI, and cloned into the vector fragment of pEVS79 that had been digested with XhoI/XbaI.

To construct pTM375, which contains the Δ*sypK* allele that removes the codons corresponding to amino acids 13-465 of SypK, the regions surrounding *sypK* were amplified from ES114 genomic DNA by PCR using primers VF_A1030-5-SalI-U/VF_A1030-5-EcoRI-L and

VF_A1030-3-EcoRI-U/VF_A1030-3-NheI-L, digested by SalI/EcoRI and EcoRI/NheI, and cloned into the vector fragment of pEVS79 that had been digested with SalI/XbaI.

Introduction of the Δ*luxPQ* and Δ*sypK* alleles into ES114 was performed as previously described for other allele exchange vectors (Miyashiro *et al.*, 2010).

Construction of KV4829, KV5972, and KV6010, which contain deletions of *luxU*, *luxQ*, and *luxP* were generated using pVAR18, pVAR29, and pVAR30, respectively, as previously described (Ray and Visick, 2012).

Chromosomal integration vectors. To construct pTM239, which contains *gfp* downstream of the *qrr1* promoter, the *qrr1* promoter region was amplified from ES114 genomic DNA by PCR using primers qrr1-prom-KpnI-U/ qrr1-prom-SpeI-L, and the product was digested with KpnI/SpeI. The *gfp* gene was isolated by digesting pTM146 with SpeI/EcoRV. The *qrr1* promoter and *gfp* fragments were ligated to the vector fragment of pEVS107 that had been digested with SpeI, made blunt with T4 DNA polymerase, and digested with XbaI. Integration of pEVS107-derived plasmids into the Tn*7* site was performed as previously described (McCann *et al.*, 2003).

IPTG-inducible plasmids. Plasmids pTM367 and pTM368 contain *sypK* (*VF_A1030*) and *sypL* (*VF_A1031*), respectively, downstream of the IPTG-inducible P_{trc} promoter. To construct pTM367, *sypK* was amplified from ES114 genomic DNA by PCR using primers sypK-KpnI-U/sypK-SalI-L, digested with KpnI/SalI, and cloned into the vector fragment of pTM214 that had been digested with KpnI/SalI. To construct pTM368, *sypL* was amplified from ES114 genomic DNA by PCR using primers sypL-KpnI-U/sypL-SalI-L, digested with KpnI/SalI, and cloned into the vector fragment of pTM214 that had been digested with KpnI/SalI.

LuxQ variant plasmids. Plasmid pVAR48 contains the wild-type *luxQ* allele tagged with the FLAG epitope. To construct pVAR48, *luxQ* was amplified from ES114 genomic DNA by PCR

using primers 1314 and 1437, digested by XhoI/KpnI, and cloned into the vector fragment of pVSV105 that had been digested with SalI/KpnI.

Plasmids pVAR50 and pVAR51 contain the A216P and H378A alleles of *luxQ-FLAG*, respectively. To construct pVAR50 and pVAR51, pVAR48 was mutagenized using the Change-IT Multiple Mutation Site Directed Mutagenesis Kit (Stratagene, La Jolla, CA, USA) with primer sets 849/1425 and 849/1426, respectively. Mutations were confirmed by sequencing.

sypK plasmid. Plasmid pVAR70 contains the wild-type *sypK* allele tagged with the FLAG epitope. To construct pVAR70, *sypK* was amplified from ES114 genomic DNA by PCR using primers 1167 and 1580, digested by XbaI/KpnI, and cloned into the vector fragment of pVSV105 that had been digested with XbaI/KpnI. The clone was confirmed by sequencing. The *sypK-FLAG* allele was subcloned from the resulting vector using SalI/BglI, and cloned into the vector fragment of pKV282 that had been cut with SalI/BamHI.

Table S1. Primers used in this study.

Primer name	Primer Sequence
luxPQ-5-XhoI-U	GGCTCGAGGTAGCGATAACTACAGATACTTTA
luxPQ-5-BamHI-L	GTGGATCCGTTGCGCATTACAACCAACCAGAA
luxPQ-3-BamHI-U	GAGGATCCGAGAACGGGTTGCCACCTTTTGAC
luxPQ-3-XbaI-L	GATCTAGAGATGTTGCGATTATGGATTTACCC
VF_A1030-5-SalI-U	AGGTCGACGCAAAAGCCATTATGGTGTTTATC
VF_A1030-5-EcoRI-L	GGGAATTCTTTAGCATGGGTGATAAAGGAAAT
VF_A1030-3-EcoRI-U	CGGAATTCTCTCATCACTTTTCCTAAAAAAGA
VF_A1030-3-NheI-L	GGGCTAGCGGATTTACTCTTTTAATTGGTTTA
qrr1-prom-KpnI-U	GAGGTACCAGCCAACACATCAAAACCTG
qrr1-prom-SpeI-L	AAACTAGTGGTCAATATACCTATTGCAG
849	CCTGTGTGAAATTGTTATCCG
1167	CATACTAAGTGCGGCCGCCTATCATTAGATGAGGTGAAACAATGAG
1314	CTGGTGTAGAACACTAGTAATG
1425	GATAATAAGATATTACCATCAAGTGTAAGTTCC
1426	TTAGCAAGAATGAGTGCTGAAATAAGAACGCCT
1437	AAAAAGGTACCTTATTTATCATCATCATCTTTATAATCCGAGTCAAAAGGTGGCAACCCG
1580	GGTACCTTATTTATCATCATCATCTTTATAATCTAGTGTTTTTCCTGTTTGTTCTTTTTTAGG
sypK-KpnI-U	CGGGTACCTTAGATGAGGTGAAACAATGAGTC
sypK-SalI-L	CGGTCGACTCATAGTGTTTTTCCTGTTTGTTC
sypL-KpnI-U	GGGGTACCGAACAAACAGGAAAAACACTATGA
sypL-SalI-L	GGGTCGACTTACTCTTTTAATTGGTTTAATGC
rpoD-qPCR-U	AGAAACCGCTCCTGATGCTGATGA
rpoD-qPCR-L	TACTGGGTCAGTTGTACGGCCAAT
litR-qPCR-U	AGGCCTAGAACAAGGCTATCTCCA
litR-qPCR-L	TAGCGACAGAGACCTGAGCGATTT
sypA-qPCR-U	AAGGTGATATGGACGCCATCGGTT
sypA-qPCR-L	AGCGCCAATACCTGATGAATCT
sypF-qPCR-U	AAGGCCCAGGAGCACAAGTTGATA
sypF-qPCR-L	GCAGCTTCTGCTTCATTTCTGGCT
sypJ-qPCR-U	CAGCTCAACTTGATCGTCTTCGCT
sypJ-qPCR-L	TCCCGCAATGAGAGCTTCAAGGAT
sypK-qPCR-U	TGGCTTTCGAAGGAATGATTGCCG
sypK-qPCR-L	ATGGCCTGAAGTAATGCTCGACCT
sypL-qPCR-U	CTTGGCATCAACGCCTCATTGGTT
sypL-qPCR-L	TCCGCCTCGACTTTGTGTTGCTAT
sypM-qPCR-U	TGCTTTACGGTGGCTTACCCTTCT
sypM-qPCR-L	ATTCGTTCGCCCACTAAAGGTCGT
sypP-qPCR-U	TTGTTGTTGGTAATGCTGGGCGAC
sypP-qPCR-L	AGCCAGAACCTGCAAGCAGTAAGT

References

McCann, J., Stabb, E. V., Millikan, D. S. and Ruby, E. G. (2003) Population dynamics of *Vibrio fischeri* during infection of *Euprymna scolopes*. *Appl Environ Microbiol* **69:** 5928-34.

Miyashiro, T., Wollenberg, M. S., Cao, X., Oehlert, D. and Ruby, E. G. (2010) A single *qrr* gene is necessary and sufficient for LuxO-mediated regulation in *Vibrio fischeri*. *Mol Microbiol* **77:** 1556-67.

Ray, V. A. and Visick, K. L. (2012) LuxU connects quorum sensing to biofilm formation in *Vibrio fischeri*. *Mol Microbiol* **86:** 954-70.
